# Methodological Caveats in the Detection of Coordinated Replay between Place Cells and Grid Cells

**DOI:** 10.3389/fnsys.2017.00057

**Published:** 2017-08-02

**Authors:** John B. Trimper, Sean G. Trettel, Ernie Hwaun, Laura Lee Colgin

**Affiliations:** ^1^Center for Learning and Memory, University of Texas at Austin, Austin TX, United States; ^2^Department of Neuroscience, University of Texas at Austin, Austin TX, United States; ^3^Institute for Neuroscience, University of Texas at Austin, Austin TX, United States

**Keywords:** hippocampus, medial entorhinal cortex, grid cells, place cells, replay, reactivation, CA1

## Abstract

At rest, hippocampal “place cells,” neurons with receptive fields corresponding to specific spatial locations, reactivate in a manner that reflects recently traveled trajectories. These “replay” events have been proposed as a mechanism underlying memory consolidation, or the transfer of a memory representation from the hippocampus to neocortical regions associated with the original sensory experience. Accordingly, it has been hypothesized that hippocampal replay of a particular experience should be accompanied by simultaneous reactivation of corresponding representations in the neocortex and in the entorhinal cortex, the primary interface between the hippocampus and the neocortex. Recent studies have reported that coordinated replay may occur between hippocampal place cells and medial entorhinal cortex grid cells, cells with multiple spatial receptive fields. Assessing replay in grid cells is problematic, however, as the cells exhibit regularly spaced spatial receptive fields in all environments and, therefore, coordinated replay between place cells and grid cells may be detected by chance. In the present report, we adapted analytical approaches utilized in recent studies of grid cell and place cell replay to determine the extent to which coordinated replay is spuriously detected between grid cells and place cells recorded from separate rats. For a subset of the employed analytical methods, coordinated replay was detected spuriously in a significant proportion of cases in which place cell replay events were randomly matched with grid cell firing epochs of equal duration. More rigorous replay evaluation procedures and minimum spike count requirements greatly reduced the amount of spurious findings. These results provide insights into aspects of place cell and grid cell activity during rest that contribute to false detection of coordinated replay. The results further emphasize the need for careful controls and rigorous methods when testing the hypothesis that place cells and grid cells exhibit coordinated replay.

## Introduction

The hippocampus is a region of the brain known to be important for spatial and declarative memory in rats, monkeys, and humans (Morris et al., 1982; Squire and Zola-Morgan, 1991; Squire, 1992; Moser et al., 1993; Vargha-Khadem et al., 1997; Maguire et al., 1998; Abrahams et al., 1999; Bachevalier and Nemanic, 2008). A subset of hippocampal neurons termed ‘place cells’ have been shown to selectively activate in discrete regions of space called ‘place fields’ (O’Keefe and Dostrovsky, 1971). During moments of sleep and quiet wakefulness, recently active place cells have been shown to reactivate in a way that can reflect recently traveled trajectories (Pavlides and Winson, 1989; Wilson and McNaughton, 1994; Skaggs and McNaughton, 1996; Nádasdy et al., 1999; Foster and Wilson, 2006; Diba and Buzsáki, 2007). Accumulating evidence supports the hypothesis that these replay events are important for learning and memory, as their disruption leads to impaired memory performance (Girardeau et al., 2009; Ego-Stengel and Wilson, 2010; Jadhav et al., 2012).

One idea for how replay contributes to learning is by facilitating memory consolidation, a process by which memories initially dependent upon the hippocampus instead become dependent upon cortical regions associated with the original sensory experience (Wilson and McNaughton, 1994; Carr et al., 2011). Specifically, during the consolidation process, replay by hippocampal neurons of the neuronal code for a particular memory is thought to be simultaneously accompanied by reactivation of the cortical neuronal code for the same experience (Jadhav et al., 2016; Rothschild et al., 2016). Across successive replay events, the neocortical representation is thought to become strengthened and stabilized such that the memory may ultimately be retrieved without hippocampal involvement. A critical piece of evidence in support of this hypothesis would be the detection of coordinated replay of representations of the same experience in hippocampal neurons and neurons in the entorhinal cortex, considering that the entorhinal cortex is the gateway between the hippocampus and sensory neocortex (Witter, 1993). Similar to place cells, grid cells in the superficial layers of the medial entorhinal cortex (MEC) also display spatially tuned receptive fields (Hafting et al., 2005). Thus, a plausible hypothesis is that grid cells also display sequential reactivation during replay events. Unlike hippocampal place cells, however, grid cells display a regularly tessellating pattern of spatial receptive fields across all environments (Hafting et al., 2005). This potentially presents a major obstacle in the detection of simultaneous replay between hippocampal place cells and MEC grid cells, as grid cell firing does not correspond uniquely to any single location.

Recently, O’Neill et al. (2017) reported evidence of significant replay by grid cells in superficial MEC layers (II/III), but further found that replay events in superficial MEC and dorsal CA1 were temporally discrete from one another with only 4% of grid cell replay events occurring in close temporal proximity to putative CA1 replay events. In a separate study, Ólafsdóttir et al. (2016) recorded simultaneously from the deep layers (V/VI) of MEC and dorsal CA1 and reported evidence of highly coordinated replay between cells in these two areas. Together, these findings present an intriguing scenario in which medial entorhinal neurons in deep layers, which receive projections from the hippocampus (Witter, 1993), reactivate content in synchrony with the hippocampus, while superficial layer MEC neurons, which project to the hippocampus (Witter, 1993), reactivate spatial representations largely independent of hippocampal activity. One implication of this physiological framework is that coordinated replay between hippocampal place cells and deep layer MEC grid cells is initiated by the hippocampus and, moreover, that replay by hippocampal place cells is rarely initiated by upstream superficial layer MEC grid cells. Replay in the superficial layers of MEC and hippocampus may therefore offer distinct functional contributions to memory. Given the aforementioned complications in assessing coordinated replay between place cells and grid cells, however, questions remain regarding the extent to which coordinated replay may have been detected spuriously. To assess this possibility, we applied analytical methods, modeled after those employed by Ólafsdóttir et al. (2016), to randomly paired MEC superficial layer grid cell and CA1 place cell ensembles recorded from distinct rats. Analyses revealed that some of the analytical methods previously used to test for coordinated replay between grid cells and place cells spuriously detected coordinated replay in surrogate recording pairs. Other statistical procedures fared much better, correctly failing to detect coordinated replay in surrogate data sets. We further compared these results to those obtained when utilizing more stringent criteria for evaluating replay in CA1, as was done by O’Neill et al. (2017). We found that including these additional requirements significantly reduced the detection of spurious results. These results lend understanding to the strengths and limitations of methods currently in use for detecting coordinated replay between the hippocampus and entorhinal cortex.

## Materials and Methods

### Subjects

Ten male Long-Evans rats weighing between 380 and 647 g (mean ± SD = 550.3 ± 77.3 g) were used for this study. In five of these rats, recordings were obtained from superficial layers of MEC. In the other five rats, recordings were obtained from the pyramidal cell layer of hippocampal subregion CA1. Recordings were performed in a single region per rat, rather than simultaneously, to maximize cell yield per region, as these data were originally collected for experimental questions distinct from those addressed by the current report. For the purpose of evaluating spuriously detected coordinated replay, recordings from distinct regions in separate animals offered a well-suited dataset, as a true co-occurrence of trajectory replay in each region was impossible, given that activity was recorded from separate animals tested at distinct times. Rats were housed on a reverse light/dark cycle (lights off from 8 to 8 pm), with behavioral sessions taking place during the dark phase. Rats were pre-trained to run on a linear track before surgical implantation of a recording device (see below). After surgery, rats were individually housed in custom-built acrylic cages (∼40 cm × 40 cm × 40 cm). Cages contained enrichment materials (e.g., plastic balls, wooden blocks, cardboard tubes). Rats recovered from surgery for at least 1 week before behavioral testing resumed and data collection began. During the data collection period, rats were food-deprived to no less than 90% of their free-feeding weight. All experiments were conducted according to the guidelines of the United States National Institutes of Health Guide for the Care and Use of Laboratory Animals under a protocol approved by the University of Texas at Austin Institutional Animal Care and Use Committee.

### Surgery

All rats were implanted with chronic electrophysiological recording devices (“drives”) containing 13–16 independently movable tetrodes. Tetrodes were constructed from 17 mm polyimide-coated platinum-iridium (90–10%) wire (California Fine Wire). Electrode tips of tetrodes designated for single unit recording were plated with platinum to reduce single channel impedances to ∼150 to 300 kW at 1 kHz. Five rats were surgically implanted with a drive above dorsal hippocampus, targeting subregion CA1 (AP: -3.8 mm, ML: 3.0 mm; 1.0 mm ventral from the brain surface on day of surgery). Another five rats were surgically implanted with a drive above MEC using the following coordinates: 0.2–0.3 mm anterior to the transverse sinus, 4.5 mm lateral from the midline, and 1 mm ventral from the surface of the brain on the surgery day. Bone screws were placed in the skull, and the screws and the base of the drive were covered with dental cement to secure the drive to the skull. Two screws in the anterior skull were connected to the recording drive to serve as an electrical ground.

### Tetrode Placement

Over the next few weeks after surgery, tetrodes were slowly lowered toward their target locations. In five rats, tetrodes were lowered to stratum pyramidale in CA1. In the other five rats, tetrodes were lowered to superficial layers of MEC. In all rats, one tetrode was used as a reference for differential recording. For hippocampal recordings, this reference tetrode was placed in an electrically quiet region at the level of the corpus callosum or higher. For MEC recordings in all but one rat, the most anterior tetrode was targeted toward the angular bundle and designated as the reference for differential recordings. Due to prominent noise on the most anterior channels in one rat, the most posterior tetrode was chosen as a reference instead. Reference tetrodes were continuously recorded against ground to ensure that they remained in electrically quiet locations across all days of recording. All recording locations were verified histologically after experiments were finished.

### Data Collection

Data were collected using the Neuralynx data acquisition system (Neuralynx, Bozeman, MT, United States). The recording drive was connected to a multichannel, unity gain headstage (HS-54, Neuralynx, Bozeman, MT, United States). The rat’s position was tracked at a 30 Hz sampling rate using light-emitting diodes (LEDs) on the headstage. The output of the headstage was conducted via two lightweight tether cables through a multichannel slip-ring commutator to a data acquisition system that processed the signals through individual 24 bit AD converters (Digital Lynx, Neuralynx, Bozeman, MT, United States). Experiments began when spikes emerged with amplitudes that were approximately five times the noise levels and when depth estimates and oscillatory activity indicated that tetrodes were in target regions (i.e., robust theta rhythms for hippocampus and MEC; prominent sharp wave-ripples for hippocampus). For spike detection, signals were digitally bandpass filtered between 600 and 6000 Hz; events that exceeded a threshold set by the experimenter (∼55–75 μV) were detected as spikes and sampled at 32 kHz. Additionally, continuous local field potential (LFP) recordings were digitally filtered in the 0.1–500 Hz band and sampled at 2000 Hz. LFPs were recorded differentially against a reference tetrode placed in an electrically silent region (see above). This reference signal was duplicated using a breakout board (MDR-50 breakout board, Neuralynx, Bozeman, MT, United States) and recorded continuously against ground. Hippocampal data used for this study were included in a previously published study (Bieri et al., 2014), and were also included in combination with some of the MEC data (i.e., from three rats) in an additional report (Zheng et al., 2015).

### Behavior

After recovering from surgery, rats resumed behavioral training, which consisted of three 10-min sessions per day on a linear track (2 m long, 10 cm wide, and 64 cm above the floor). Rats were trained to run back and forth on the track, as described previously (Bieri et al., 2014). Rats were rewarded with small pieces of sweet cereal or cookies at both ends of the track. Before data acquisition began, rats were trained on the track for at least 3 days to ensure environmental familiarity. Each recording session was preceded and followed by ∼10-min rest sessions. During each rest session, rats were placed in a towel-lined, elevated flower pot. Between 5 and 12 recording days were conducted for each animal (see **Table 1**). Analyses were restricted to rest periods from behavioral sessions, as rats’ position and speed could not be assessed during rest bouts in the flower pot.

**Table 1:** Descriptive statistics including number of sessions, cells, and events per animal for CA1 and medial entorhinal cortex (MEC) recordings.

Brain region	Rat ID	# Sessions used	Cell yield (range)	# Events/session
**CA1**				
	Rat #1	3	20–26	78.3 ± 16.8
	Rat #2	6	20–24	118.3 ± 26.49
	Rat #3	1	22	15 ± 0
	Rat #4	4	29–40	87.0 ± 21.1
	Rat #5	2	24–28	39.0 ± 11.0
**MEC**				
	Rat #1	5	6–9	50
	Rat #2	4	5–8	50
	Rat #3	1	5	50
	Rat #4	14	4–7	50
	Rat #5	10	4–9	50


*Bouts of grid cell firing were chosen at random, with a limit of 50 events per session set for each grid cell rat.*

### Results, Statistics, and Data Analyses

Data were analyzed using custom software written in MATLAB (MathWorks, Natick, MA, United States), unless indicated otherwise. Primary analysis functions are available from authors upon request. Specific analysis methods are described in detail below. Results are reported in figures and text as mean ± SEM, unless indicated otherwise.

### Spike Sorting and Single Unit Classification

Spike sorting was performed offline using graphical cluster-cutting software (MClust; A.D. Redish, University of Minnesota, Minneapolis). Spikes were clustered manually in two-dimensional projections of the multidimensional parameter space. Autocorrelation and cross-correlation functions were additionally used to identify single units. In hippocampal recordings, putative place cells were distinguished from putative interneurons on the basis of spike width, average firing rate, and bursting properties (Fox and Ranck, 1981; Harris et al., 2000; Henze et al., 2000; Frank et al., 2001). To be classified as a place cell, CA1 units were required to exhibit peak in-field firing rates of at least 1 Hz, and a place field length of at least 20 cm. In MEC, we considered only the activity of spatially modulated grid cells (Hafting et al., 2005), which were identified by calculating a ‘gridness score’ as described in Sargolini et al. (2006). Each gridness score was compared to a bootstrapped distribution of gridness scores, constructed using 2,000 repetitions in which spike times were shifted by a random amount, keeping the inter-spike intervals fixed while changing the spike positions. Cells with gridness scores greater than the 95th percentile of the bootstrapped distribution of scores were classified as grid cells. Grid fields were defined as portions of the linear track, at least three contiguous spatial bins in length (i.e., 6 cm), where the smoothed rate map (see below) exhibited a firing rate of greater than 0.01 Hz.

### Place Cell and Grid Cell Firing Rate Maps

Place and grid cell firing rate maps were constructed in the following manner. A 25 cm portion was excluded from each end of the 198 cm linear track to remove reward locations where rats paused. These portions of the track were used to detect putative replay events (see below). The rats’ positions along the remaining track length were then linearized. The track was divided into 2 cm bins, and spike rates were measured for each bin. The resultant rate map was smoothed with a Gaussian kernel (5 cm standard deviation; 30 cm width). Separate rate maps were constructed for inbound and outbound directions.

### Place Cell Replay Detection and Evaluation

Putative replay events were detected based on multi-unit activity (MUA), following a procedure described by Pfeiffer and Foster (2013) and also used by Ólafsdóttir et al. (2016) and O’Neill et al. (2017). MUA activity at the ends of the track, when rats were most likely to be stationary, was sorted into 1 ms bins and smoothed with a Gaussian kernel (5 ms standard deviation; 30 ms width). Putative replay events were detected as periods of time in which MUA exceeded the mean rate by three standard deviations. Onsets and offsets of putative replay events were detected as the times before and after the peak MUA when activity returned to the mean. Event onsets and offsets were then re-calibrated to the earliest and latest spike time, respectfully, within the event bounds. Events less than 40 ms in duration were discarded. For CA1, only events in which at least 15% of the place cells were active were included.

To analyze reactivation of spatial sequence memory during putative replay events, spiking activity during CA1 putative replay events was sorted into 5 ms bins. Bayesian decoding was then employed to generate the posterior probability matrix for each putative replay event. The posterior probability matrix is the two-dimensional array describing the probability of the animal’s presence in each 2 cm spatial bin given the observed pattern of spikes at a given time. Two posterior probability matrices were generated for each event, one for each running direction. A line of best fit was established for each posterior probability matrix with ordinary least squares linear regression (MATLAB polyfit) using the spatial bins corresponding to maximal probability values at each time point. A fit score was calculated, for each direction, as the average probability per time bin within ±30 cm from the best fit line. The fit score for each direction was then compared to a chance distribution (see below) to evaluate the statistical significance of the putative replay event and to determine which direction (i.e., inbound vs. outbound) best corresponded to the putative replay event.

Chance distributions were established for each direction by shifting the rate map for each single unit by a random value between one and the number of track bins minus one. After shifting the rate maps, trailing ends were wrapped around to ensure equal length rate maps for each unit. This procedure was repeated 100 times for each event and direction. For each iteration, a fit score was calculated as described in the previous paragraph. The fit scores for the non-shifted data, for each direction, were then percentile ranked against their corresponding chance distributions, and only events ranked greater than 80% of the shifted fit scores (i.e., *p* < 0.2) were accepted for further analysis. The direction for each event was assigned based on which direction, inbound or outbound, was associated with a lower *p*-value. The significance of putative replay events was also evaluated against an alpha level of 0.025 (i.e., 97.5th percentile) to assess the impact of more stringent inclusion criteria.

In addition to utilizing the replay evaluation procedure described above, modeled after Ólafsdóttir et al. (2016), we also assessed place cell replay following a more rigorous procedure detailed by O’Neill et al. (2017). We then contrasted the frequency at which coordinated replay between grid cells and place cells was detected using each of these two approaches to assess the impact of different replay detection criteria. An additional criterion enforced by O’Neill et al. (2017) was the comparison of putative replay events to a second chance distribution in which within-event spike times for each unit were shuffled relative to one another. Arrays of spike times for each unit within putative replay events were shuffled 100 times by a random amount between 5 ms and the length of the event minus 5 ms. This procedure shuffles the temporal relationship between units but preserves the relative spike timing within units. As with the spatial shuffling procedure described above, a fit score was calculated for each shuffle relative to the original line of best fit. All replay events with fit scores surpassing the 80th percentile of both the temporal and spatial shuffle distributions of fit scores (i.e., alpha = 0.20) were accepted as replay and subjected to further analysis. In line with O’Neill et al. (2017), we also evaluated the detection of coordinated replay when the line of best fit imposed over the place cell posterior probability matrix was required to traverse at least 12 cm, or exhibit a slope of at least 200 cm/s, and compared these results to those obtained without this requirement.

### Evaluating Coordination between Place Cell Events and Grid Cell Firing

CA1 place cell replay events were randomly paired with duration-matched epochs of grid cell firing (i.e., a time window within or around grid cell firing epochs) recorded from separate animals. Grid cell firing epochs were initially selected as bouts of time in which at least one grid cell fired at least one action potential while the rats were at the end of the track and moving less than 5 cm/s. Given that these lenient selection criteria could lead to identification of a very high number of epochs from each rat, we restricted the maximum number of grid cell firing epochs to 50 epochs per experimental session. We also assessed coordinated replay when a 5 spike minimum was required for each grid cell firing epoch, as was done by O’Neill et al. (2017). Coordination between place cell replay events and grid cell firing epochs was evaluated using two separate methods described in detail below.

#### Method 1: Spatial Coherence (**Figure 2**)

The first evaluation method relied upon a metric termed spatial coherence, derived as follows, and was utilized by both Ólafsdóttir et al. (2016) and O’Neill et al. (2017). First, grid cell firing during the randomly selected firing epochs was decoded using Bayesian probability statistics, as was done with CA1 replay events. The line of best fit from a randomly paired place cell event was then imposed over the grid cell posterior probability matrix. The spatial coherence between place cell events and grid cell firing epochs was calculated as the summed probability within 0.5*x* cm of the place cell event’s line of best fit normalized by the number of time bins, with *x* defined as the average grid field size for each animal. Note that use of this 0.5*x* spatial window follows Ólafsdóttir et al. (2016), whereas O’Neill et al. (2017) employed a non-varying window of 11.73 cm. The impact of differences in spatial window size is explored further below.

The statistical significance of spatial coherence scores was assessed by comparing the observed values to three different shuffle distributions, in line with the procedure utilized by Ólafsdóttir et al. (2016). First, each grid cell event was paired with 100 other randomly selected place cell events (Event Shuffle in **Figures 2B,C**). For each of these 100 place cell events, the line of best fit for each posterior probability matrix (see above) was imposed over the grid cell event’s posterior probability matrix but extended or shortened to match the duration of the grid cell event. For each of these grid cell-place cell pairings, the spatial coherence was assessed as described above. Second, grid cell rate maps were spatially shifted 100 times for each event, preserving the order of spatial bins within the rate map and shifting the rate map in its entirety, by a random amount between 10 spatial bins and the length of the track minus 10 spatial bins (Spatial Shuffle in **Figures 2B,C**). Again, for each shuffle, the spatial coherence was then assessed. Third, the array of spike times for each unit within the grid cell firing epoch was shuffled by a random amount between 5 ms and the length of the event minus 5 ms, thereby shuffling the temporal relationships between units but preserving the relative spike timing within the array of spike times for each unit (Temporal Shuffle in **Figures 2B,C**). For each temporal shuffle, spatial coherence was assessed as before.

The observed distribution of coherence scores was then compared to each of these shuffle/chance distributions using the following procedure modeled after analyses described by Ólafsdóttir et al. (2016). The observed data were bootstrapped 10,000 times (subsampled with replacement), and the area under the cumulative distribution curve (i.e., the sum of the cumulative distribution) was assessed for each bootstrap. Difference scores between the area under the curve (AUC) for the shuffle distributions and actual data were calculated for each of the 10,000 bootstraps and the 95% confidence intervals were assessed based on these difference scores for each shuffle type. A result was deemed significant if the confidence interval for the distribution of AUC values from all 10,000 bootstraps did not contain 0 (rightmost column of **Figure 2B**). This entire spatial coherence assessment and statistical evaluation procedure was repeated 1,000 times, and the proportion of times a significant result was obtained with each shuffling procedure was assessed.

#### Method 2: Event Map Correlations (**Figure 3**)

As in Ólafsdóttir et al. (2016), a second statistical evaluation procedure was also employed to assess coordinated replay between place and grid cell firing. For each grid cell within each randomly selected MEC firing epoch, an ‘event map’ was constructed by aligning the grid cell spike times, relative to the beginning of the epoch, with the line of best fit from a randomly paired place cell replay event. Each grid cell spike time was assigned to a particular spatial bin based on the ordinate position of the line at that particular time, and the event map was constructed by compiling the firing rates across all of the spatial bins.

The correlation between this event map and each grid cell’s actual rate map was assessed, and each observed correlation value was ranked against two shuffle distributions constructed as follows. First, rate maps were spatially shifted by all possible distances between one bin and the total number of spatial bins (Spatial Shuffle in **Figure 3**). The relative order of bins was preserved within each rate map. For each shift, the correlation between the shifted map and the event map was reassessed, producing a chance distribution of correlation values for each firing epoch. Second, the array of grid cell spike times within each randomly selected firing epoch was randomly shifted 100 times by a value between 0 ms and the duration of the paired place cell event, thereby changing the temporal relationship between paired place and grid cells but preserving the order of spikes within grid cell spike trains (Temporal Shuffle in **Figure 3**). After each shift, an event map was constructed as described above, but using the newly shifted spike times. The correlation between this event map and a grid cell’s actual rate map was assessed for each shuffle, producing a chance distribution of correlation values for the 100 event maps constructed from the temporally shifted spike trains.

The observed correlation values for each original event map and rate map pairing (one for each grid cell within each grid cell firing epoch) were then percentile ranked relative to that unit’s/epoch’s Spatial and Temporal Shuffle distributions, ultimately producing a distribution of percentile ranks across all grid cells and grid cell firing epochs. This distribution of percentile ranks was then statistically evaluated against chance (i.e., 50%) using Wilcoxon’s signed-rank test. As with the spatial coherence method, this event map procedure was repeated 1,000 times, and the proportion of times a significant result was obtained with each shuffling procedure was assessed.

## Results

### Initial Replay Detection

Grid cells (6.01 ± 0.47 per session, per rat; 201 total) and place cells (25.6 ± 1.38 per session, per rat; 410 total) were recorded from five MEC and five CA1 animals across an average of 6.80 ± 2.31 and 3.20 ± 0.86 days of linear track sessions, respectively. **Table 1** presents additional details for the number of cells, sessions, and events detected per CA1 and MEC animal. Initial replay detection in CA1 revealed 1,386 candidate replay events. Evaluating each of these candidate events against chance distributions in which spike times were shuffled left 1,347 significant events remaining (*p* < 0.20) (685 inbound; 662 outbound) (see Materials and Methods for details regarding replay detection and evaluation of significance).

**FIGURE 1:**
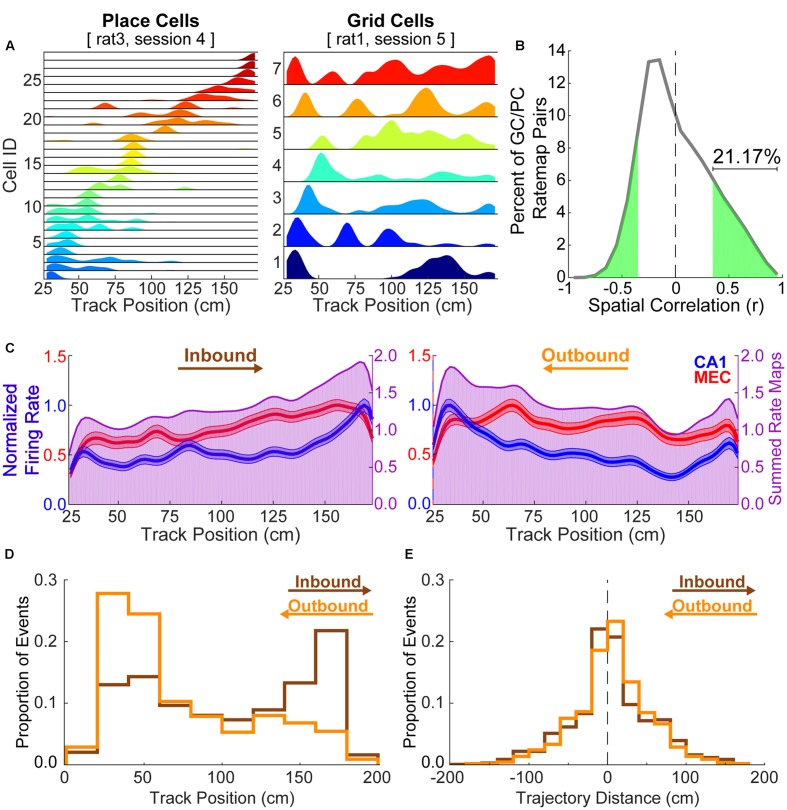
Grid cell rate maps are often correlated with place cell rate maps by chance. **(A)** Example place cell (left) and grid cell (right) rate maps. Grid cells often show multiple, broad spatial receptive fields on a linear track while place cells typically exhibit a single, relatively restricted spatial receptive field. **(B)** 21.17% of paired grid cell and place cell rate maps from different rats are significantly positively correlated by chance. **(C)** Average rate maps for each track running direction and region (CA1 = blue; MEC = red, left axes) show that place cells disproportionately represent the track ends relative to other locations. The summed average rate map (purple, right axes) facilitates visualization of spatial overlap in each region’s average rate map. **(D)** Histograms show the distribution of start locations for each CA1 replay trajectory by replay direction (inbound = brown; outbound = orange). Trajectory start locations beyond the range of track positions considered when formulating rate maps (i.e., within 25 cm of the ends of the 198 cm track) were observed when the y intercept for the line of best fit was outside of the spatial range of the posterior probability matrix (i.e., 25–173 cm). Note that replayed trajectories, as defined following the procedure described in section “Materials and Methods,” tend to begin at ends of the track opposite of what one might expect given the calculated replay direction. **(E)** Replayed trajectories often traversed only a small portion of the linear track (inbound = 4.49 ± 1.99 cm; outbound = 5.28 ± 1.84 cm).

**Figure 1A** shows example rate maps for CA1 place cells and MEC grid cells taken from separate animals. For each animal, cells recorded during a single experimental session are shown. As is apparent in these examples, individual place cells tended to exhibit only one, spatially restricted place field, as described previously (e.g., O’Keefe and Dostrovsky, 1971; Wilson and McNaughton, 1993, but see Henriksen et al., 2010; Oliva et al., 2016 for place cells recorded across a greater proximodistal extent of CA1 than in the present study). In contrast, grid cells tended to exhibit multiple firing fields on the linear track, consistent with prior reports (e.g., Hafting et al., 2008). Multiple spatial receptive fields were detected in 82.11 ± 1.11% of grid cells per animal, whereas only 2.34 ± 0.60% of place cells per animal exhibited more than one place field. This multi-field feature of grid cells presents an obstacle to analyses of coordinated replay between place cells and grid cells, as does the likelihood of significant positive spatial correlations between place cell and grid cell rate maps. Indeed, 21.17% of grid cell and place cell pairings exhibited statistically significant positively correlated spatial maps, despite being recorded from separate animals (**Figure 1B**). Place cells in particular tended to disproportionately represent the ends of the linear track (**Figure 1C**). This factor likely contributed to place cell replay events in both directions preferably beginning at track ends (**Figure 1D**). In the majority of cases, trajectories replayed by CA1 place cells constituted a relatively small portion of the track (**Figure 1E**).

### Results Using the Spatial Coherence Method

**FIGURE 2:**
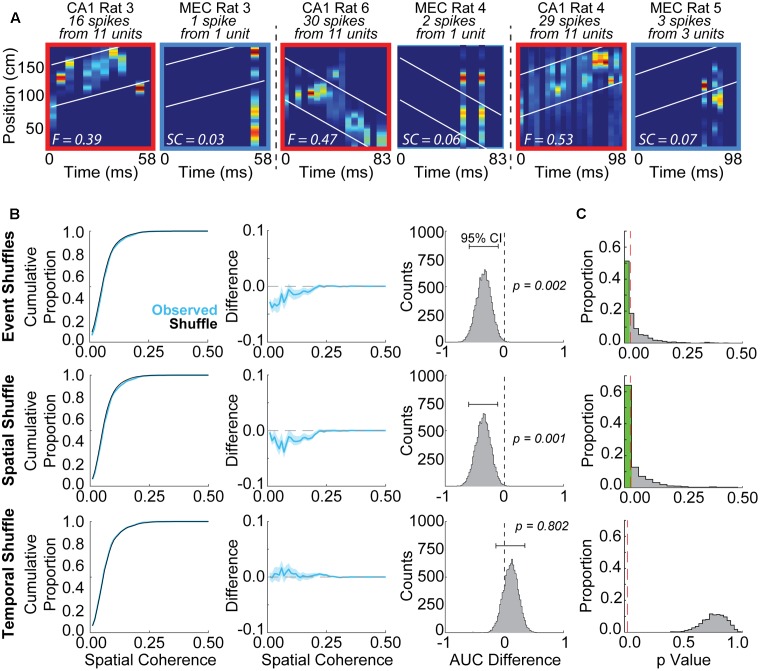
Grid cells and place cells recorded from different rats exhibit spuriously significant spatial coherence using some shuffling methods but not others. **(A)** Examples of posterior probability matrices for randomly paired place cell replay events and grid cell firing epochs. Rat numbers, as well as cell and spike counts, are indicated at top of each panel. White lines indicate ±30 cm from the line of best fit applied to the CA1 posterior probability matrix. Fit (F) or spatial coherence (SC) scores are indicated by the white text within each panel. **(B)** Example results from a single iteration using the Spatial Coherence method to evaluate coordinated replay between grid and place cells. A lower area under the curve was observed for the bootstrapped ‘Observed’ data distribution (blue, mean ± SD) relative to the shuffle distribution (black) for Event Shuffles (top row) and Spatial Shuffles (middle row), but not Temporal Shuffles (bottom row). This observation indicates spuriously greater spatial coherence scores for the randomly paired data relative to the event and spatial shuffled data, but not the temporal shuffled data. The difference between the two curves (Observed – Shuffle) is plotted in the middle column. The right column displays the distribution of differences between the area under the curve for the shuffle and each bootstrap of the Observed data (brackets indicate 95% confidence intervals for this example iteration). **(C)** Results are shown from 1,000 iterations of the Spatial Coherence statistical evaluation procedure using randomly paired grid cell and place cell firing epochs. 56.9% of iterations were spuriously detected as statistically significant when compared to Event Shuffle distributions (top row). 60.5% of iterations were spuriously detected as significant when compared to Spatial Shuffle distributions (middle row). No iterations were found to be significant when compared to Temporal Shuffle distributions (bottom row). Red dashed lines indicate an alpha level of 0.025.

The high prevalence of positive correlations between place cell and grid cell maps from different animals raises the possibility that coordinated replay between grid cells and place cells may be detected spuriously. To determine the extent to which coordinated replay between place cells and grid cells may be spuriously detected, we randomly paired CA1 replay events with epochs of MEC grid cell firing recorded from separate rats and employed evaluation methods utilized previously by others (Ólafsdóttir et al., 2016). **Figure 2A** presents several examples of randomly paired place cell replay events and grid cell firing epochs. The previously described (Ólafsdóttir et al., 2016) spatial coherence method (see Materials and Methods) was used to evaluate how well locations represented during the place cell replay events matched with the locations represented by the grid cell firing epochs. Despite low grid cell spike counts, spatial coherence scores were often found to be significantly greater than chance when compared to a subset of shuffle distributions.

Illustrative results from a single iteration of the spatial coherence method for each of the three shuffle approaches are presented in **Figure 2B**. A lower proportion of low spatial coherence scores are apparent for the ‘Observed’ distribution relative to the Event Shuffle distribution in this example iteration (**Figure 2B**, left two columns, top row). We calculated difference scores, corresponding to the AUC of the cumulative proportion plot from each bootstrap of the Observed data minus the corresponding AUC of the shuffle distribution. For this representative iteration, the 95% confidence interval for the distribution of difference scores was negative and did not overlap with zero, indicating greater spatial coherence in the Observed distribution than in the chance distribution (**Figure 2B**, right column, top row). This example is consistent with most results from 1,000 iterations of this analysis procedure (**Figure 2C**, top row). Specifically, when observed spatial coherence scores were compared to distributions in which place cell events were randomly shuffled using the Event Shuffle method, spuriously significant results were obtained on 56.9% of iterations (**Figure 2C**, top). A similar result is revealed for the example comparison between the Observed data and corresponding Spatial Shuffle distribution in **Figure 2B** (middle row), in which grid cell rate maps are randomly shifted 100 times by a random number of spatial bins. Here too, the 95% confidence interval for the distribution of AUC differences scores in this example does not overlap with zero, indicating significantly higher spatial coherence in the Observed data compared to the Spatial Shuffle distribution. Across 1,000 iterations, spuriously significant results such as in this example were obtained on 60.5% of iterations (**Figure 2C**, middle row). These Event Shuffle and Spatial Shuffle methods correspond to two of the methods used to demonstrate coherent grid cell and place cell replay in a previous report (see Figure 2B in Ólafsdóttir et al., 2016). These results suggest that simultaneously occurring grid cell and place cell activity during rest may often be erroneously characterized as significantly coordinated replay when Event Shuffle and Spatial Shuffle methods are used to assess replay.

A representative example of a comparison between Observed data and a corresponding Temporal Shuffle distribution is shown in **Figure 2B** (bottom row). In this example, the 95% confidence interval for the distribution of AUC difference scores overlaps with zero, indicating significantly higher spatial coherence was not found in the Observed data compared to the Temporal Shuffle distribution (**Figure 2B**, bottom row). Indeed, no iterations returned spuriously significant findings when tested against spike-time shuffled distributions (i.e., Temporal Shuffles; **Figure 2C**, bottom row). These results indicate that temporally shuffling spike times produced suitable comparison distributions, in contrast to inappropriate comparison distributions produced by shuffling event pairs and rate maps.

### Results Using the Event Map Correlations Procedure

**FIGURE 3:**
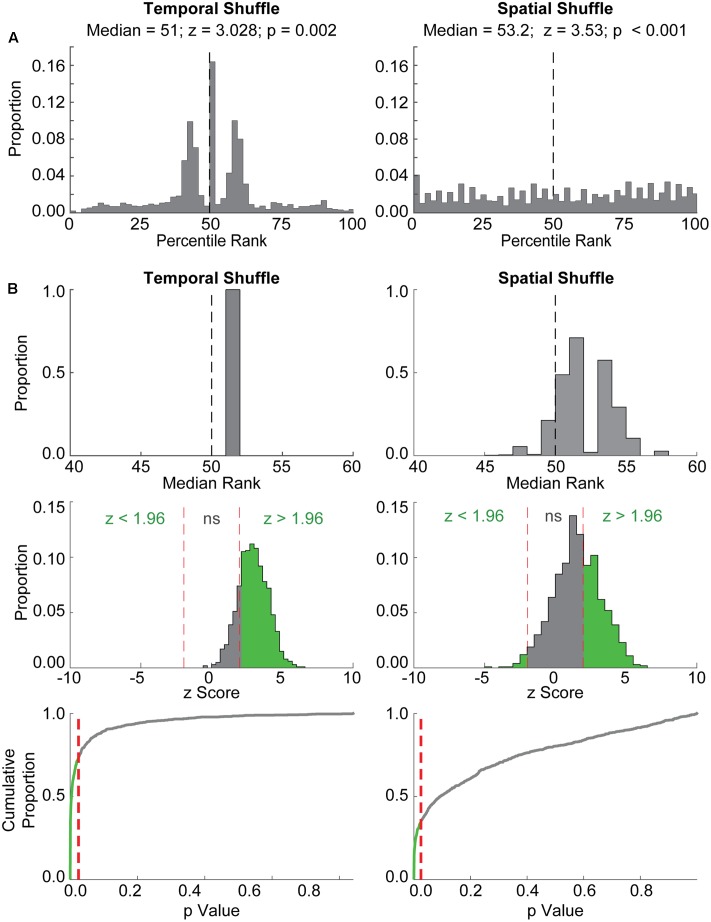
The event map method also detects spuriously significant coordination between place cell replay events and random epochs of grid cell firing from different rats. **(A)** Example results from a single iteration of the ‘event map’ evaluation procedure are shown. Histograms depict the distribution of ranks for each Observed correlation between randomly paired grid and place cell firing epochs relative to the distribution of either temporally shuffled (left) or spatially shuffled (right) data. The median of each distribution, along with the *z*-statistic and *p*-value resulting from the Wilcoxon Signed Rank test for these examples, are shown at top. The vertical dashed line at 50% represents the median rank that would be expected by chance. Although the distribution of Temporal Shuffle ranks was centered around ∼50% and the Spatial Shuffle distribution was relatively uniform, both comparisons with Observed correlations were significant using the Wilcoxon Signed Rank Test. **(B)** Results from 1,000 iterations of the event map procedure are shown. Histograms in the top row depict the distributions of median ranks from each of the 1,000 iterations relative to Temporal Shuffle and Spatial Shuffle distributions. Note that many of the median ranks are greater than 50, indicating that correlations between randomly paired grid cell and place cell firing epochs would often be characterized as significant. The middle row shows the distribution of *z*-scores, corresponding to Wilcoxon’s signed rank test-statistic (W), with the proportion of significant results indicated in green. *P*-values for each iteration are depicted in the bottom row as cumulative distributions. Red dashed lines indicate an alpha level of 0.025. The green portion of the line corresponds to the proportion of spuriously significant results observed.

We next assessed the extent to which another previously used statistical evaluation procedure (i.e., “event ratemap” procedure in Ólafsdóttir et al., 2016) returned spuriously significant results. In this procedure, the likelihood of detecting correlations between grid cell rate maps and ‘event maps’ aligned to place cell replay events (see Materials and Methods) was assessed. We performed 1,000 iterations of this procedure using randomly paired place cell replay events and grid cell firing epochs. Example results for a single iteration are shown in **Figure 3A**. Plotted in each panel is the distribution of ranks obtained when comparing the observed correlation between a grid cell rate map and its corresponding event map to correlations between the grid cell rate map and event maps created by temporally shuffling the grid cell spike train (left) or spatially shuffling the grid cell rate map (right). A high rank would be presumed to indicate strong alignment between place cell replay and grid cell firing, whereas a rank near 50% would be presumed to indicate a near chance level of coordination between grid cells and place cells. Although the distribution of ranks relative to the Temporal Shuffle distribution was centered at approximately the 50th percentile (**Figure 3A**, left), a Wilcoxon signed-rank test returned a significant *p*-value of 0.002 for this example. Similarly, despite the relatively uniform distribution of ranks for the Observed data relative to the Spatial Shuffle distribution (**Figure 3A**, right), a Wilcoxon signed-rank test again returned a significant *p*-value (i.e., <0.001). Thus, in this example, coordination between a place cell replay event and a randomly paired grid cell firing epoch from a different animal was erroneously found to be significant. The results obtained in this example were similar to findings from 1,000 iterations of this ‘event map’ analysis procedure (**Figure 3B**). The distribution of median percentile ranks assessed relative to the Temporal (left) and Spatial (right) Shuffle chance distributions from each of the 1,000 iterations reveals that all of the median percentile ranks relative to the Temporal Shuffle, and the majority of the median percentile ranks relative to the Spatial Shuffle, were greater than the 50th percentile. Indeed, across 1,000 iterations, place cell replay events and randomly paired grid cell firing epochs were spuriously found to be significantly coordinated (*p* ≤ 0.025) on 73.6% of iterations for the Temporal Shuffle method (**Figure 3B**, left, bottom two rows) and 33.5% of iterations for the Spatial Shuffle method (**Figure 3B**, right, bottom two rows). This result indicates that creating ‘event maps’ for grid cell spike trains using co-occurring place cell replay events often leads to spurious detection of coordinated replay and thus is not a rigorous method for determining whether coordinated replay occurs between place cells and grid cells.

### Varying Parameters

We next set out to determine whether varying analysis parameters would alleviate some of the issues inherent in the afore-described analysis methods. Initially, a distance of ±30 cm relative to the line of best fit for the place cell posterior probability matrix was employed in all analyses to evaluate significance of place cell replay. Likewise, a distance of ±half of each animal’s average grid field size was used to calculate spatial coherence scores in the above-described spatial coherence method. However, our track was a third of the linear length (200 cm) of the track employed by Ólafsdóttir et al. (2016) (600 cm), raising the question of whether reducing these two distance parameters to scale with the smaller track length would reduce the number of spuriously detected significant results. To address this question, each of these parameters was systematically altered, and, for each new combination of parameters, the results of 1,000 iterations using both analysis methods described above were evaluated. **Table 2** presents these findings. Lowering the distance around the line of best fit used to evaluate replay in CA1 from ±30 cm to ±10 cm led to a small to moderate increase in the percent of iterations returning spurious results for all shuffle distributions and methods tested. When the distance from the place cell event’s best fit line used for calculating spatial coherence scores was lowered from one-half of the average grid field size to one-quarter of the average grid field size, the detection of spuriously coordinated replay between place cells and grid cells decreased by only a small to moderate degree. Thus, reducing these distance parameters to account for a shorter track length failed to meaningfully reduce the spurious detection of coordinated replay events.

**Table 2:** The results of 1,000 iterations of each statistical evaluation procedure using various combinations of distance parameters.

		Method 1: PC line fit to GC PPM			Method 2: Event/rate map correlation	
			
PC Fit Distance	GC fit distance	Event shuffle	Spatial shuffle	Temporal shuffle	Temporal shuffle	Spatial shuffle
±30 cm	Half of grid field	56.9%	60.5%	0.0%	73.6%	33.5%
	Quarter of grid field	41.6%	60.3%	0.0%	–	–
±10 cm	Half of grid field	77.6%	75.3%	0.0%	86.5%	36.5%
	Quarter of grid field	63.7%	65.9%	0.0%	–	–


*Percentages indicate the proportion of 1,000 iterations for each set of parameters that returned spuriously significant results. Either 30 cm or 10 cm was used for evaluating the significance of place cell replay events, and either ± one-half or ± one-quarter of each rat’s average grid field size, was employed when evaluating spatial coherence. PC, place cell; GC, grid cell; PPM, posterior probability matrix.*

Following Ólafsdóttir et al. (2016), we then asked how employing a lower alpha level when assessing CA1 replay would impact the results. Specifically, we lowered the alpha level used to evaluate the significance of CA1 candidate replay events from 0.200 to 0.025. We then repeated 1,000 iterations of each analysis procedure. Mixed results were observed. The proportion of iterations returning spuriously significant findings was reduced using the Event Shuffle spatial coherence (56.9 to 32.7%), Spatial Shuffle spatial coherence (60.5 to 37.8%), and Spatial Shuffle event map (33.5 to 21.8%) methods. However, the proportion of iterations detecting significantly correlated replay was greater for the Temporal Shuffle spatial coherence procedure (73.6 to 85.7%). These findings indicate that simply applying a more stringent alpha level for replay detection is not sufficient to mitigate concerns raised by the present results.

### Results Observed Using Methods of O’Neill et al. (2017)

To ask how a more rigorous evaluation of CA1 replay significance would impact results, we repeated 1,000 iterations of each of the coordinated replay analysis procedures described above after evaluating replay using the methods employed by O’Neill et al. (2017). Specifically, in addition to comparing putative CA1 replay events to a Spatial Shuffle distribution, replay was also evaluated against a Temporal Shuffle distribution. A shorter distance around the line of best fit imposed over the CA1 replay posterior probability matrix was also employed (i.e., 12 cm vs. 30 cm), as was a minimum replay trajectory (12 cm distance) or slope (200 cm/s). Enforcing these criteria greatly reduced the number of spurious findings when coordination was evaluated against Event and Spatial Shuffle distributions following the Spatial Coherence evaluation procedure, while spurious results obtained relative to Temporal Shuffle distributions remained at 0.0%. **Table 3** shows a summary of these results. Similar results were obtained when the minimum trajectory distance/slope criterion was not enforced. Combined with the above-described finding regarding reduced fit distance (see Varying Parameters), these results indicate that the factor that most strongly reduces spurious findings is the inclusion of Temporal Shuffle comparisons in place cell replay detection methods.

**Table 3:** The results of 1,000 iterations of each statistical evaluation procedure using different replay evaluation procedures and minimum grid cell spike counts.

	Method 1: PC line fit to GC PPM			Method 2: Event/rate map correlation	
		
Replay evaluation method	Event shuffle	Spatial shuffle	Temporal shuffle	Temporal shuffle	Spatial shuffle
Ólafsdóttir et al. Approach (Version 1)	56.9%	60.5%	0.0%	73.6%	33.5%
(1) Replay evaluated against spatial shuffle only					
(2) No minimum trajectory enforced					
(3) One grid cell spike minimum					
Event Counts: PC, *n* = 1347; GC, *n* = 1700					

O’Neill et al. Approach (Version 1)	8.2%	9.2%	0.0%	70.9%	13.9%
(1) Replay evaluated against both spatial and temporal shuffle					
(2) Minimum trajectory enforced					
(3) One grid cell spike minimum					
Event Counts: PC, *n* = 907; GC, *n* = 1700					

O’Neill et al. Approach (Version 2)	8.4%	8.3%	0.0%	71.2%	13.3%
(1) Replay evaluated against both spatial and temporal shuffle					
(2) No minimum trajectory enforced					
(3) One grid cell spike minimum					
Event Counts: PC, *n* = 1311; GC, *n* = 1700					

O’Neill et al. Approach (Version 3)	34.9%	5.1%	0.0%	0.5%	5.0%
(1) Replay evaluated against both spatial and temporal shuffle					
(2) Minimum trajectory enforced					
(3) Five grid cell spike minimum					
Event Counts: PC, *n* = 907; GC, *n* = 719					


*For each set of parameters, percentages indicate the proportion of 1,000 iterations that returned spuriously significant results. The data displayed here correspond to those described in the text in Section “Results Observed Using Methods of O’Neill et al. (2017).” PC, place cell; GC, grid cell; PPM, posterior probability matrix.*

Despite a reduction in spurious findings using the Spatial Coherence procedure with more rigorously evaluated replay in CA1, detection of coordinated replay between grid cell and place cells using the event map correlations procedure was still found to be spuriously significant on 70.9 and 13.9% of iterations relative to Temporal and Spatial Shuffle distributions, respectively (**Table 3**). To ask how enforcing a minimum grid cell spike count would impact these results, considering that O’Neill et al. (2017) required at least five grid cell spikes per grid cell firing epoch and Ólafsdóttir et al. (2016) required only one, we applied a five grid cell spike minimum condition and ran another 1,000 iterations for both analysis procedures. Enforcing a minimum grid cell spike count requirement reduced spurious findings using the event map correlation procedure considerably (i.e., 0.5 and 5.0% using Temporal and Spatial Shuffle distributions, respectively). This minimum grid cell spike requirement also reduced spurious findings observed against Spatial Shuffle distributions using the Spatial Coherence procedure (from 9.2 to 5.1%). Paradoxically, incorporating a minimum grid cell spike requirement increased the detection of spuriously coordinated replay when the Event Shuffle distribution was used (from 8.2 to 34.9%). For the Temporal Shuffle version of the spatial coherence method, the percent of spuriously coordinated replay that was detected remained at 0.0%. In summary, spurious results are least likely to be observed using the spatial coherence method when CA1 replay is detected using both temporal and spatial shuffle distributions and a minimum grid cell spike count is enforced. The Event Shuffle component of the spatial coherence procedure is the most problematic analysis with regards to returning spurious findings.

## Discussion

The present study employed previously utilized statistical procedures for detecting coordinated replay across MEC grid cells and hippocampal place cells (Ólafsdóttir et al., 2016; O’Neill et al., 2017) to determine the extent to which coordinated replay is spuriously detected between randomly paired place cell and grid cell recordings from separate animals. Analyses revealed a range of performance reliability across the various shuffle distributions and methods employed, with some statistical procedures reliably returning no spuriously significant results and others returning spuriously significant findings on a majority of iterations. The application of more rigorous replay detection criteria and minimum grid cell spike requirements greatly reduced the percent of spurious findings. Thus, whereas some of the previously employed approaches may be appropriate for the detection of coordinated replay, other approaches require revisions to prevent the potentially spurious detection of coordinated replay.

Hippocampal replay has been proffered as one mechanism supporting the systems-wide consolidation of declarative memory (Walker and Stickgold, 2006; Carr et al., 2011), with hippocampal replay hypothetically serving as the impetus for simultaneous cortical reactivation of the broader memory trace. Important empirical support for this hypothesis, therefore, is the detection of coordinated replay between hippocampal and cortical ensembles. Though some support for coordinated replay has begun to emerge from studies utilizing dual recordings in hippocampus and prefrontal cortex (Peyrache et al., 2009; Jadhav et al., 2016), or hippocampus and deep layers of MEC (Ólafsdóttir et al., 2016), the question of whether analogous representations are reactivated in extra-hippocampal regions exclusively in response to hippocampal initiation remains open. For example, Jadhav et al. (2016) reported that neuronal activity in the rodent prefrontal cortex (PFC) was indeed synchronously modulated by hippocampal replay events. However, Jadhav et al. (2016) also found that the content being reactivated in PFC during these synchronous events was not exclusively related to the hippocampally reactivated content. Likewise, in a separate report from the same laboratory, Rothschild et al. (2016) reported that patterned activity in the auditory cortex preceded and predicted hippocampal replay. Most recently, O’Neill et al. (2017) reported evidence of temporally independent replay in grid cells recorded from the superficial layers of the MEC (II/III) and place cells recorded from dorsal CA1. In sum, though some data are beginning to accumulate in support of the idea that hippocampal replay can drive cortical reactivation of analogous content, data are also emerging in support of the idea that cortical replay can occur independent of the hippocampus. Moreover, it remains unclear whether hippocampal replay drives, or occurs in response to, cortical reactivation.

An important question for improving upon the existing experimental approaches is to ask which specific features contribute to spurious detection of coordinated replay across structures. First, it is important to note that place cell and grid cell rate maps from different animals running in similar environments were found to be significantly positively correlated approximately 20% of the time (**Figure 1B**). This finding lends some understanding as to why shuffling rate maps (i.e., Spatial Shuffles) in the event map procedure produced distributions in which correlation values were often lower than those obtained for randomly paired place cell and grid cell events. Second, an inherent problem when assessing grid cell and place cell replay is the regularly repeating spatial receptive fields of grid cells. As these neurons possess multiple fields that are not restricted to a particular spatial region, their posterior probability matrices are likely to display high probabilities in multiple, regularly repeating areas of space, thus increasing the odds of observing high spatial coherence using the methods employed herein. Though place cells in distal CA1 often exhibit multiple place fields (Henriksen et al., 2010; Oliva et al., 2016), these fields tend not to be regularly tessellating and thus potentially pose less of an issue than the regularly spaced receptive fields of grid cells. This issue of multiple receptive fields may be at least partially alleviated in data sets containing large cell and spike counts, because the grid cell posterior probability matrix should more accurately reflect a rat’s location with higher numbers of simultaneously recorded neurons. Indeed, requiring a minimum number of grid cell action potentials per grid cell firing epoch reduced the detection of spuriously coordinated replay in the current report. Also, Bayesian decoded real-time positions were more accurate when all MEC cells, not just grid cells, were included in analyses in a previous study (O’Neill et al., 2017). Finally, attempts to identify replay in grid cells would benefit from additional experimental controls. One such control involves testing whether replay events detected in recordings from a particular ensemble of grid cells similarly correlate with activity in a subsequently tested novel environment. This control is necessary to determine the extent to which activity characterized as replay is explained by pre-existing correlations in MEC grid cell networks (Burak and Fiete, 2009; Couey et al., 2013; Pastoll et al., 2013).

Grid cells in MEC exhibit regularly repeating spatially tuned receptive fields (Hafting et al., 2005), presenting a challenge to decoding efforts aimed at revealing the spatial locations represented by firing patterns of grid cells. Here, we present results of analyses performed on hippocampal place cells and MEC grid cells recorded from separate animals in which spuriously significant coordination was detected on a substantial proportion of iterations with a subset of previously implemented methods (Ólafsdóttir et al., 2016). Utilizing the more rigorous methods of a subsequent report (O’Neill et al., 2017) greatly reduced spurious detection of coordinated replay. The results lend understanding to the limitations inherent in previously applied methods and emphasize the necessity of including rigorous controls when testing the hypothesis that MEC grid cells and hippocampal place cells exhibit coordinated replay.

## Author Contributions

JT conducted all analyses with guidance from LC and input from ST and EH. ST and EH collected grid cell recordings. JT and LC wrote the manuscript. All authors engaged in valuable discussions. The authors also acknowledge the Texas Advanced Computing Center (TACC) at The University of Texas at Austin for providing data storage resources that have contributed to the research described within this paper. URL: http://www.tacc.utexas.edu.

## Conflict of Interest Statement

The authors declare that the research was conducted in the absence of any commercial or financial relationships that could be construed as a potential conflict of interest. The reviewer KD declared that he co-hosts a research topic with one of the authors LC and the absence of any other collaboration. The handling Editor has been informed and ensures that the process met the standards of a fair and objective review.
